# Effect of chilled eye drops on postoperative pain sensation after phototherapeutic keratectomy: Randomised controlled clinical trial

**DOI:** 10.1111/aos.70033

**Published:** 2025-11-12

**Authors:** Carolin Marion Kolb‐Wetterau, Julian Bucur, Marvin Lucas Biller, Petra Dávidová, Klemens Paul Kaiser, Kleopatra Varna‐Tigka, Ingo Schmack, Thomas Kohnen

**Affiliations:** ^1^ Department of Ophthalmology Goethe‐University Frankfurt am Main Germany; ^2^ Department of Ophthalmology University Medical Center of the Johannes Gutenberg University Mainz Germany

**Keywords:** chilled eye drops, cooled eye drops, phototherapeutic keratectomy, postoperative pain, postoperative therapy

## Abstract

**Purpose:**

To analyse pain sensation after phototherapeutic keratectomy (PTK) using chilled eye drops or drops at room temperature during the early postoperative period.

**Methods:**

Our randomised controlled, parallel‐group study conducted in the Department of Ophthalmology, Goethe‐University, Frankfurt (Main), Germany, with blinded participants and outcome assessors included patients undergoing PTK. Postoperatively, eye drops in one group were chilled and in the other group at room temperature (warm). Patients completed pain questionnaires six times on the first 3 postoperative days. Pain intensity was primarily assessed by means of the numerical rating scale (NRS) at 8 a.m. on Day 1. Secondary outcomes included pain on the visual analogue scale (VAS), sensory qualities of pain, overall pain intensity, epiphora, foreign body sensation and additional need for analgesics.

**Results:**

Fifty‐one patients were analysed in the chilled group and 49 in the warm group. Median NRS and VAS on Day 1 were 2 (range: 0–8) and 13 (0–76) in the chilled group and 1 (0–8) and 4 (0–79) in the warm group, respectively (*p* = 0.11 and 0.10). On Day 2, values were 2 (0–7) and 14 (0–52) in the chilled group and 2 (0–10) and 19 (0–99) in the warm group (*p* = 0.34 and 0.82). There was no significant difference in secondary outcomes. Additional painkillers on Day 1 were required by 29% and 18%, respectively (*p* = 0.23).

**Conclusion:**

Using chilled eye drops following PTK does not reduce pain compared with eye drops at room temperature in the early postoperative period.

## 1 INTRODUCTION

Most patients experience postoperative pain after corneal surface ablation which is often severe and extremely stressful. The management of postoperative pain is of great importance and while several studies have evaluated pain following photorefractive keratectomy (PRK), studies on pain after phototherapeutic keratectomy (PTK) remain scarce. Apart from pain, discomfort including tearing and foreign body sensation is a relevant side effect of surface ablation (Sobas et al., 2015).

In order to develop adequate concepts of postoperative pain management, the underlying molecular mechanisms need to be understood. The cornea is the most densely innervated tissue with 7000 nerve endings per square millimetre (Müller et al., 2003). The fibres are derived from long posterior ciliary nerve branches that originate from the trigeminal nerve. A high density of nerve fibres is located within the corneal epithelium, in the subepithelial nerve fibre plexus and the anterior stroma (Marfurt et al., 2010). Seventy per cent of sensory afferent fibre endings are nociceptors. These slow conducting fibres respond to extreme temperature, mechanical damage, exogenous chemical irritants or inflammatory mediators released by damaged corneal cells or resident inflammatory cells. Twenty per cent are mechano‐receptors and respond to mechanical stimulation (Belmonte et al., 2004; Garcia, de Andrade, et al., 2016; Garcia, Torricelli, et al., 2016).

During PRK and PTK, the corneal epithelium and its basal membrane are removed, followed by photoablation of Bowman's layer and the anterior stroma. First of all, laser application induces an increase in temperature which stimulates the nociceptors (Garcia, de Andrade, et al., 2016; Garcia, Torricelli, et al., 2016). Secondly, exposure of nerve fibre terminations leads to intense pain, foreign body sensation and tearing, among others, due to spontaneous firing and modified responsiveness of polymodal nociceptors (Gallar et al., 2007). Mechanical irritation due to blinking also stimulates corneal nerve fibre endings. Finally, inflammation plays an important role in pain sensation. Pain modulators are, for example, prostaglandins and neuropeptides (Garcia, de Andrade, et al., 2016; Garcia, Torricelli, et al., 2016).

Multiple strategies have been evaluated in order to decrease pain after surface ablation, for example, pharmacological options, the insertion of a bandage contact lens or the use of cooled balanced salt solution (BSS) to reduce the temperature increase due to laser application (Förster et al., 1997; Kitazawa et al., 1999; Steigleman et al., 2023). To date, no study has evaluated differences between chilled eye drops and eye drops at room temperature in the postoperative period after PRK or PTK. Pain perception and inflammation may be more pronounced in PRK than in PTK due to the longer procedure time and greater ablation depth.

To the best of our knowledge, there is only one trial dealing with postoperative pain after PTK (Förster et al., 1997). Therefore, research needs to be conducted to evaluate pain and discomfort after PTK. We hypothesised that chilled eye drops would alleviate pain after PTK compared with eye drops at room temperature. Pharmacological approaches may demonstrate side effects. In contrast, using eye drops with different temperatures is not likely to implicate additional risks. The use of chilled eye drops might be a simple and economic management of postoperative pain relief. The aim of our study was to analyse if there is a difference in postoperative pain after PTK using either chilled or eye drops at room temperature.

## 2 METHODS

The study was conducted according to the CONSORT statement and the pain‐specific supplement (Gewandter et al., 2019; Moher et al., 2010). It is a randomised controlled, parallel‐group study performed at the Department of Ophthalmology, Goethe‐University, Frankfurt (Main), Germany. Patients were randomly assigned to one of two parallel groups in a 1:1 ratio to receive either chilled or room temperature eye drops.

Eligible participants were all adults aged 18 or older who were referred to our clinic for PTK between May 2023 and June 2025. Exclusion criteria were corneal pathologies except for the diagnosis that led to the indication for PTK, prior ocular surgery, active or chronic ocular infection, history of herpetic keratitis or uveitis, chronic pain anywhere, analgesics as long‐term medication, pregnancy or lactation. Patients who had prior PTK on the other eye were also excluded. Adequate German language ability was mandatory to answer the questionnaire.

Informed consent was given by all participants. The study protocol was reviewed and approved by the local university institutional ethics committee. Tenets of the Declaration of Helsinki were followed throughout the study. The trial was registered in the German Clinical Trials Register (number DRKS00031747).

Surgery was performed by one experienced surgeon (T.K.). After manual epithelial abrasion with a hockey stick spatula, the excimer laser (SCHWIND AMARIS 750S, SCHWIND eye‐tech‐solutions GmbH, Kleinostheim, Germany) was used for corneal ablation with a depth of 5 or 10 μm. Afterwards, the ocular surface was rinsed with cold BSS having a temperature of 4–8°C and dexamethasone and ofloxacin eye drops were instilled. At the end, a soft bandage contact lens (PureVision, Bausch + Lomb, Rochester, NY, USA), which had been refrigerated for 2 h, was applied.

For allocation (chilled eye drops or eye drops at room temperature) of the participants, block‐randomisation by a computer‐generated random number list with an allocation ratio of 1:1 was prepared by an investigator not involved in the implementation process. Allocation was noted on a sheet of paper and put in a sealed and opaque envelope labelled with a study identification number (ID) to ensure allocation concealment. The assignment schedule was locked. After being enrolled in the study, participants consecutively received a study ID. Name and date of birth were written on the corresponding envelope. It was handed over to the nursing staff who opened the envelope and applied the assigned eye drops. During the entire period, participants and outcome assessors were blinded to the allocation. Participants did not know where the eye drops were stored and were not allowed to touch the drop container. Healthcare providers who were informed about the allocation were not involved in any other step of the study.

The trial was conducted during the regular inpatient stay of three nights after PTK. Participants were invited to complete the same questionnaire regarding current pain sensation at 6 different time points. Only pain sensation in the operated eye should be considered. The first questionnaire should be completed directly after arriving on the ward and the second one 2 h postoperatively. After filling in the second form, patients got metamizole 500 mg orally and application of eye drops was started according to the following schedule: dexamethasone (Dexa EDO, 1.3 mg/dL, Dr Gerhard Mann, Berlin, Germany) and ofloxacin eye drops (Floxal EDO, 3 mg/dL, Dr Gerhard Mann, Berlin, Germany) four times a day at 7 a.m., 12 p.m., 5 p.m. and 10 p.m. as well as preservative‐free artificial tears (Corneregel Fluid EDO, Dr Gerhard Mann, Berlin, Germany) hourly between 7 a.m. and 10 p.m. Patients were randomly assigned to receive either chilled eye drops or eye drops at room temperature (20–25°C). Chilled eye drops were stored in the fridge at 4–8°C. The general postoperative therapeutic regimen, that is, drugs and time of administration, was equal in both groups. There was no difference in the appearance of the single‐use drop containers. The interval between eye drop application and completing the questionnaires should be longer than 15 min. The third questionnaire was planned at 6 h postoperatively and the fourth, fifth and sixth one at 8 a.m. on Days 1, 2 and 3 after surgery. Pain was assessed at 8 a.m. to ensure a standardised comparison, as the postoperative eye drop regimen begins at 7 a.m., ensuring all patients had received the same number of drop applications prior to assessment on that day. Further administration of analgesics was not intended but participants were instructed to ask for rescue medication if necessary. The standard rescue medication was metamizole 500 mg orally up to four times a day. Ibuprofen 400 mg orally was administered in case of an allergy.

The primary end point was pain sensation on a numeric rating scale (NRS) from 0 to 10 (0 = no pain, 10 = worst imaginable pain) at 8 a.m. on the first postoperative day. Secondary end points were assessed on the above‐mentioned six points in time and included pain on the NRS and visual analogue scale (VAS; 100 mm horizontal line; 0 = no pain, 100 = worst imaginable pain), 5 sensory qualities of pain experience (based on the Short‐Form McGill Pain Questionnaire; throbbing, stabbing, burning, tender, shooting; 0 = none, 1 = mild, 2 = moderate, 3 = severe), the overall intensity of total pain experience (based on the Short‐Form McGill Pain Questionnaire; 0 = no pain, 1 = mild, 2 = discomforting, 3 = distressing, 4 = horrible, 5 = excruciating), epiphora (0 = none, 1 = mild, 2 = moderate, 3 = severe), foreign body sensation (0 = none, 1 = mild, 2 = moderate, 3 = severe) and the additional need for analgesics.

To detect a difference in pain experience of 1 unit on the NRS (expected SD of 1.5) with a two‐sided significance level of 5% and a power of 90%, a sample size of 49 participants per group was necessary (98 participants in total).

Data were analysed in case the questionnaire on Day 1 was completed, even if data for other individual timepoints were missing. Analyses were conducted using an available‐case approach. No imputation of missing data was performed. Analysis was carried out using IBM SPSS Statistics software (version 29.0.2.0). Data measured on an ordinal scale (sensory qualities, overall intensity, epiphora, foreign body sensation) were assumed to have interval‐scale properties. The Kolmogorov–Smirnov test was used to test for normal distribution. Since all scoring systems were not normally distributed, variables were reported as medians with ranges and 95% confidence intervals (CI), and the non‐parametric Mann–Whitney *U*‐Test was used for comparisons between groups. To compare proportions of participants between both groups, the chi‐square test with Yates' continuity correction was used and the relative risk (RR) was calculated. A *p*‐value of <0.05 was considered to be statistically significant. Bonferroni correction was used for multiple comparisons. This resulted in an adjusted significance threshold of *p* < 0.008. No interim analyses for efficacy or futility were done.

In addition to the primary analyses, subgroup analyses of NRS, VAS and overall pain intensity were conducted for age strata, sex and ablation depth. No adjustment for multiple testing was performed, as these analyses were exploratory and intended to be hypothesis‐generating.

## 3 RESULTS

**FIGURE 1:**
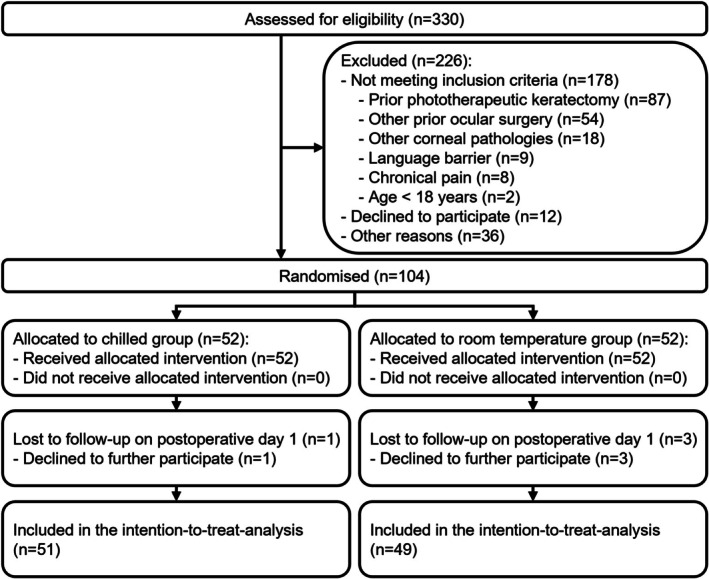
Participant flow diagram.

In total, 104 patients were enrolled. The participant flow diagram is shown in Figure 1. One patient in the chilled eye drops group and three patients in the group getting eye drops at room temperature (warm) declined further participation in the study on Day 1. In the final analysis, there were 51 participants (28 male and 23 female) in the chilled group and 49 (23 male and 26 female) in the warm group. All participants who underwent randomisation were analysed in the groups to which they were originally allocated. The baseline characteristics are shown in Table 1. No adverse events, particularly cold‐related effects such as cold‐induced discomfort or reflex tearing, were reported.

**TABLE 1:** Baseline demographic and clinical characteristics.

	Chilled eye drops (*n* = 51)	Eye drops at room temperature (*n* = 49)
Age	47.7 ± 14.8 years (range: 26–80 years)	51.4 ± 15.0 years (range: 19–85 years)
Sex (male)	28 (55%)	23 (47%)
Ethnic origin		
Asian	0 (0%)	2 (4%)
Caucasian	51 (100%)	47 (96%)
Laterality (right eye)	25 (49%)	21 (43%)
Diabetes mellitus	3 (6%)	2 (4%)
Indication for phototherapeutic keratectomy		
Epithelial basement membrane dystrophy (EBMD)	25 (49%)	28 (57%)
Recurrent corneal erosion without EBMD	17 (33%)	8 (16%)
Salzmann nodular degeneration	6 (12%)	9 (18%)
Corneal scar	3 (6%)	3 (6%)
Other	0 (0%)	1 (2%)
Ablation depth (μm)		
5	43 (84%)	40 (82%)
10	8 (16%)	9 (18%)

### 3.1 NRS, VAS and overall pain intensity

**FIGURE 2:**
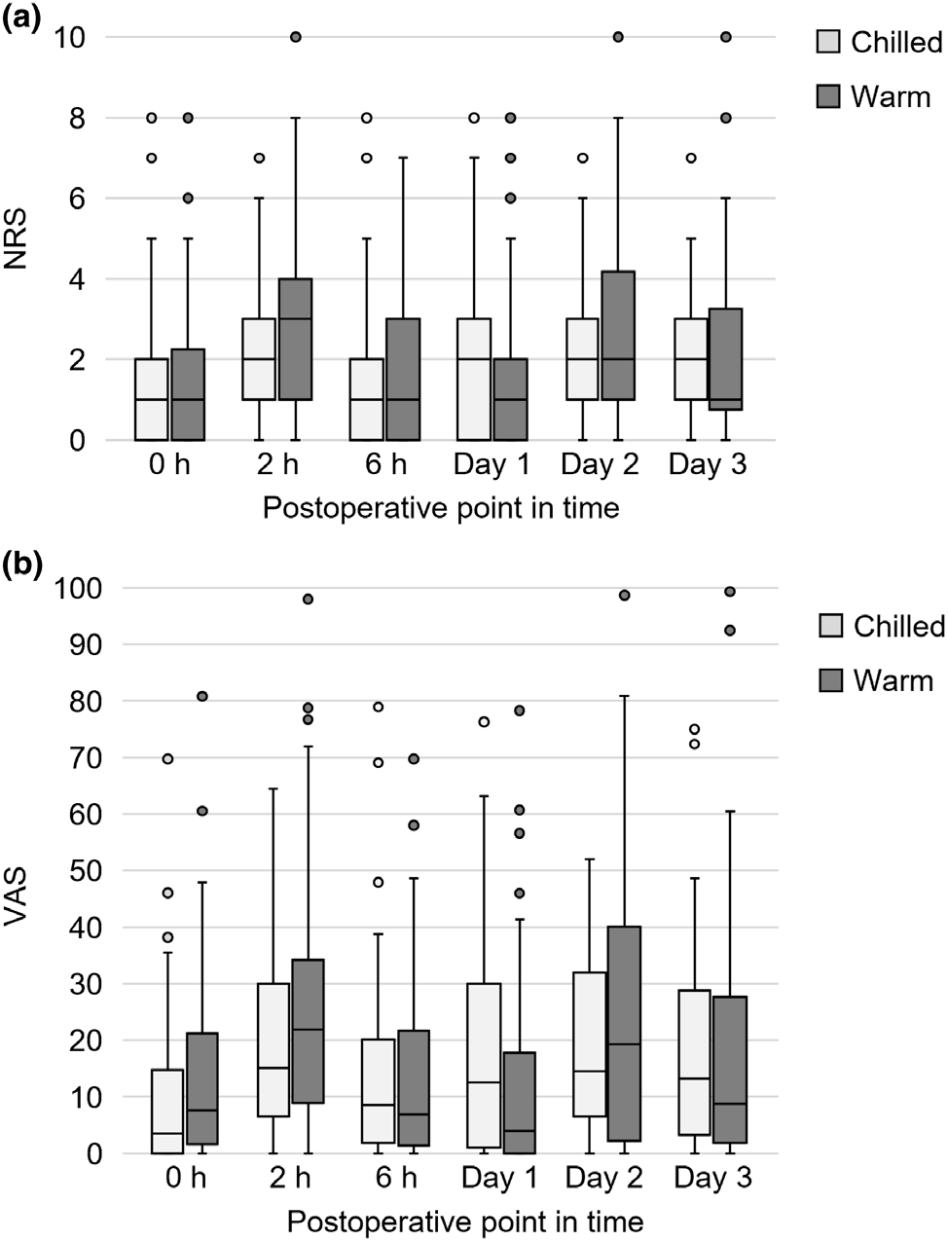
Pain sensation on the numeric rating scale (NRS) and visual analogue scale (VAS). Boxes represent the interquartile range (IQR), with the horizontal line indicating the median. Whiskers show the minimum and maximum values within 1.5× IQR. Outliers are displayed as individual points. No intergroup difference reached statistical significance.

**FIGURE 3:**
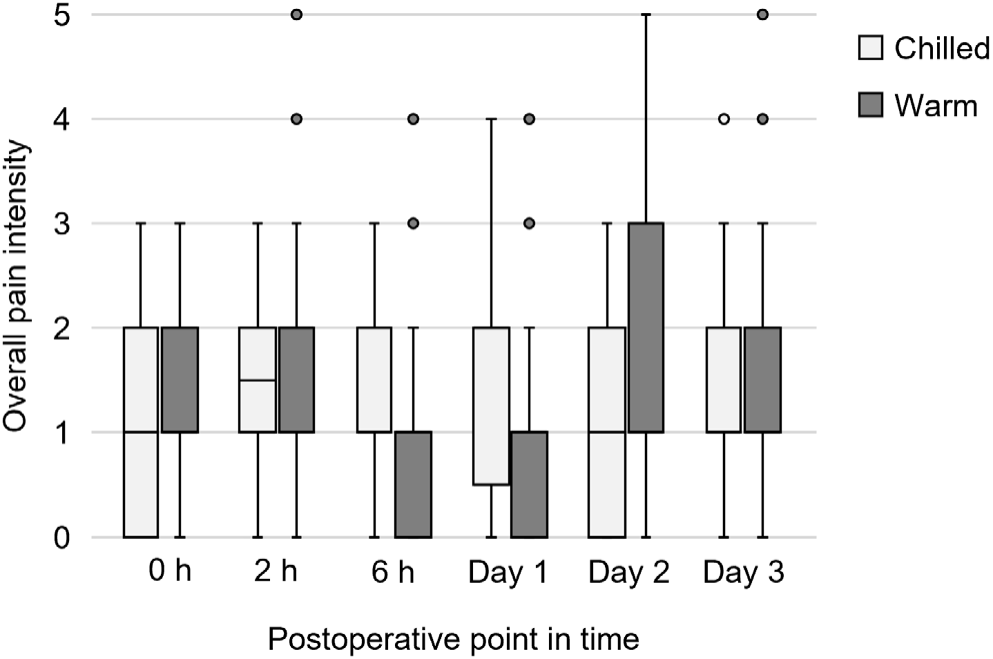
Overall intensity of total pain experience. 0 = no pain, 1 = mild, 2 = discomforting, 3 = distressing, 4 = horrible, 5 = excruciating. Boxes represent the interquartile range (IQR), with the horizontal line indicating the median. Whiskers show the minimum and maximum values within 1.5× IQR. Outliers are displayed as individual points. No intergroup difference reached statistical significance.

Figures 2 and 3 illustrate pain sensation on the NRS, VAS and the overall intensity at all time points. Intergroup differences did not reach statistical significance. Median NRS, VAS and overall pain intensity on Day 1 were 2 (range: 0–8; 95% CI: 1.3–2.4), 13 (0–76; 12.3–23.7) and 2 (0–4; 1.10–1.72) in the chilled eye drops group and 1 (0–8; 0.9–2.2), 4 (0–79; 7.7–20.2) and 1 (0–4; 0.80–1.45) in the warm eye drops group, respectively (*p* = 0.11, 0.10 and 0.11, Mann–Whitney *U*‐test) with Hodges–Lehmann estimators of 0 (95% CI: 0–1), 4 (0–10) and 0 (0–1). On Day 2, NRS, VAS and overall pain intensity values were 2 (0–7; 1.6–2.7), 14 (0–52; 14.7–24.2) and 1.5 (0–3; 1.16–1.80) in the chilled group and 2 (0–10; 2.1–3.7), 19 (0–99; 18.1–34.1) and 2 (0–5; 1.41–2.15) in the warm group (*p* = 0.34, 0.82 and 0.32) with Hodges–Lehmann estimators of 0 (−1–0), −1 (−11–6) and 0 (−1–0).

Exploratory subgroup analyses were performed to assess whether sex, age or ablation depth were associated with differences in NRS, VAS and overall pain intensity. The results are shown in Table 2. In the male subgroup, overall pain intensity was higher in the chilled group compared with the warm group on Day 1 (*p* = 0.04). On Day 2, female patients reported higher NRS pain scores in the warm group (*p* = 0.02). The subgroup analyses by age and ablation depth did not reveal any significant differences in pain outcomes.

**TABLE 2:** Subgroup analyses.

		NRS			VAS			Overall pain intensity			Additional need for analgesics		NRS ≥2	
		Chilled	Warm	*p*	Chilled	Warm	*p*	Chilled	Warm	*p*	Chilled	Warm	Chilled	Warm
*Day 1: Postoperative*														
Age	<40 years, *n* = 17/14	2 (1.3–3.8)	1.5 (0.9–3.5)	0.71	16 (10.9–36.6)	8 (4.5–33.3)	0.50	2 (1.1–2.4)	1 (0.7–2.1)	0.30	41%	14%	71%	50%
	40–60 years, *n* = 23/23	1 (1.0–2.2)	0 (0.3–2.1)	0.08	10 (8.2–21.0)	2 (2.3–20.3)	0.09	1 (0.9–1.6)	1 (0.7–1.7)	0.47	26%	17%	48%	22%
	>60 years *n* = 11/12	1 (1.0–2.1) *n* = 11	1 (0.7–2.1)	0.88	5 (9.6–22.2)	6 (6.4–19.9)	0.81	1 (0.8–1.5)	0.5 (0.5–1.0)	0.41	18%	25%	36%	17%
Sex	Male, *n* = 28/23	2 (1.2–2.8)	1 (0.4–1.7)	0.07	13 (10.3–28.1)	2 (2.7–16.5)	0.06	2 (1.0–1.9)	1 (0.5–1.2)	*0.04*	29%	13%	54%	22%
	Female, *n* = 23/26	2 (1.0–2.6)	1 (0.9–3.0)	0.62	13 (8.8–24.4)	9 (6.8–28.1)	0.54	1 (0.9–1.8)	1 (0.8–1.9)	0.78	30%	23%	52%	35%
Ablation depth	5 μm, *n* = 43/40	2 (1.5–2.7)	1 (0.9–2.4)	0.96	16 (13.9–26.8)	6 (7.6–22.4)	1.00	2 (1.2–1.8)	1 (0.8–1.6)	0.89	35%	20%	60%	33%
	10 μm, *n* = 8/9	0 (−0.3–1.5)	0 (−0.8–2.8)	0.10	0 (−3.0–11.9)	1 (−7.3–25.4)	0.06	1 (0.1–1.9)	1 (0.2–1.6)	0.11	0%	11%	13%	11%
*Day 2: postoperative*														
Age	<40 years	3 (2.8–4.4)	4.5 (2.9–6.9)	0.24	30 (21.4–38.5)	43 (26.4–71.1)	0.13	2 (1.8–2.7)	2 (1.7–3.6)	0.65	43%	46%	100%	83%
	40–60 years	1 (0.8–1.9)	1 (1.2–3.4)	0.35	9 (6.4–17.7)	6 (7.8–29.1)	0.95	1 (0.6–1.5)	1 (1.0–1.9)	0.19	26%	35%	39%	48%
	>60 years	2 (1.2–2.2)	2 (1.6–2.6)	0.65	13 (13.9–25.1)	16 (12.9–23.1)	0.88	1.5 (0.9–1.5)	1.5 (1.2–1.8)	0.58	20%	25%	55%	58%
Sex	Male	2 (1.6–3.2)	1.5 (1.1–2.7)	0.50	17 (13.6–28.9)	6 (6.9–22.7)	0.25	1.5 (1.0–1.9)	1 (1.0–1.9)	0.91	36%	30%	64%	50%
	Female	2 (1.3–2.5)	3 (2.5–5.1)	*0.02*	12.5 (10.4–23.9)	31 (21.5–48.6)	0.06	1.5 (1.1–2.0)	2 (1.5–2.7)	0.09	38%	40%	59%	68%
Ablation depth	5 μm	2 (1.6–2.7)	2 (2.1–4.0)	0.67	13 (13.7–24.5)	17 (16.5–36.5)	0.85	1 (1.1–1.8)	2 (1.4–2.3)	0.61	38%	36%	62%	58%
	10 μm	2.5 (0.5–3.5)	2 (0.9–4.1)	0.41	22 (16.0–27.2)	19.5 (4.9–43.7)	0.87	2 (0.9–2.8)	2 (0.7–2.3)	0.20	29%	33%	63%	67%

*Note*: Data are medians and 95% confidence intervals. *p*‐values are derived from Mann–Whitney *U*‐tests. Italic values indicate statistically significant results (*p* < 0.05).

Abbreviations: NRS, numerical rating scale; VAS, visual analogue scale.

### 3.2 Sensory qualities of pain experience

There was no statistical significance in any of the comparisons of sensory qualities of pain experience between the study groups. Median values of sensory qualities of pain (throbbing, stabbing, burning, tender, shooting) were 0, 0, 1, 1, 0 in the chilled group on Day 1 and 0, 0, 0, 1, 0 in the warm group. Addition of values of the 5 qualities revealed a median of 2 (0–10; 2.03–3.61) and 1 (0–10; 1.67–3.22), respectively (*p* = 0.42). On Day 2, median values of the sensory qualities of pain were 0, 0, 1, 1, 0 in the chilled group and 0, 1, 1.5, 1, 0 in the warm group. Median values of the addition were 3 (0–12; 2.84–4.44) and 3.5 (0–14; 3.07–5.10), respectively (*p* = 0.74).

### 3.3 Epiphora and foreign body sensation

**FIGURE 4:**
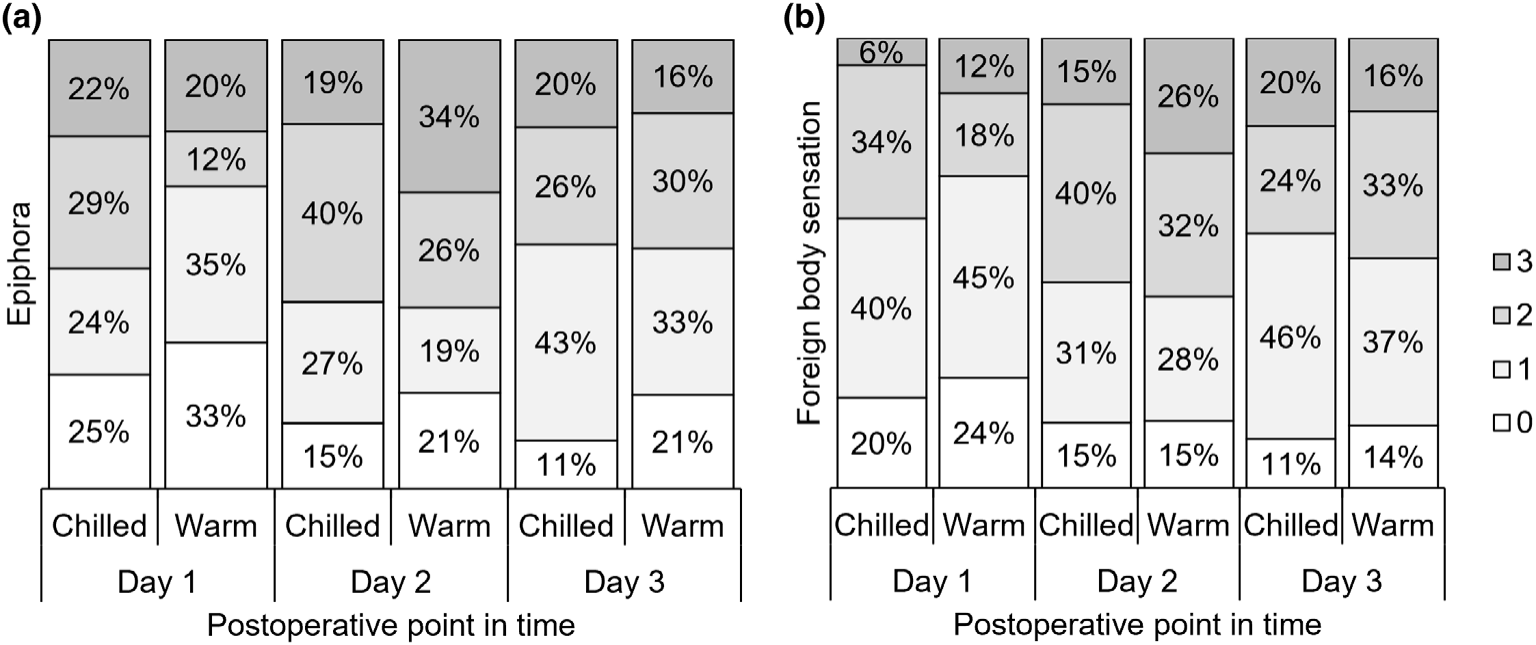
Epiphora and foreign body sensation. 0 = none, 1 = mild, 2 = moderate, 3 = severe. No intergroup difference reached statistical significance.

On Day 1, median epiphora was 2 (0–3; 1.15–1.79) in the chilled group and 1 (0–3;0.88–1.53) in the warm group (*p* = 0.22). Values of epiphora on Day 2 were 2 (0–3; 1.35–1.90) in the chilled group and 2 (0–3; 1.39–2.06) in the warm group (*p* = 0.64). Median foreign body sensation was 1 (0–3; 1.01–1.51) in the chilled group and 1 (0–3; 0.91–1.46) in the warm group on Day 1 (*p* = 0.52), and 2 (0–3; 1.27–1.81) and 2 (0–3; 1.39–1.97) on Day 2, respectively (*p* = 0.48). Details of epiphora and foreign body sensation are illustrated in Figure 4. All intergroup differences did not reach statistical significance.

### 3.4 Reported pain during the study period

On the first postoperative day, there was a significant difference between both groups, with 53% having pain in the chilled group and 29% in the warm group (*p* = 0.023, chi‐square test; RR = 1.83). These values were 62% and 60% on Day 2, respectively (*p* = 0.97; RR = 1.04). Eighty‐eight per cent of patients reported pain (NRS ≥2) during the entire observation period in the chilled group as well as in the warm group. Severe pain (NRS ≥7) was reported in 4% with chilled eye drops and 6% with eye drops at room temperature on Day 1 (*p* = 0.96; RR = 0.64), in 2% and 11% on Day 2 (*p* = 0.18; RR = 0.19) and in 16% and 22% at least at one visit during the study period (*p* = 0.54; RR = 0.70).

### 3.5 Additional need for painkillers

Twenty‐nine per cent in the chilled group and 18% in the warm group required additional painkillers on Day 1 (*p* = 0.23; RR = 1.61), 37% and 35% on Day 2 (*p* = 0.82; RR = 1.04) and 49% and 43% in the whole postoperative period, respectively (*p* = 0.55; RR = 1.14).

The median time to first rescue medication was 21 h in the chilled group and 32 h in the warm group. In the chilled group, a single administration of additional analgesics on Day 1 was sufficient in 62% of cases, compared to 75% in the warm group. A second dose was required in 31% and 25% of cases, respectively. On Day 2, a single administration was sufficient in only 40% of patients in the chilled group and 44% in the warm group. In the chilled group, 7% of patients required three or more administrations, whereas in the warm group this applied to 38%.

On both Days 1 and 2, the required dosage for most patients varied from 500 to 1000 mg of metamizole per day in the chilled group, and from 500 to 1500 mg of metamizole or 400 to 1800 mg of ibuprofen per day in the warm group. On Day 1, one patient in each group required a higher dosage and a combination of metamizole and ibuprofen. On Day 2, the same patient in the chilled group and another patient in the warm group required a higher analgesic dosage.

The pattern of analgesic use differed between the two groups on Day 1. In the chilled eye drops group, the highest demand for painkillers occurred between 6 a.m. and 12 p.m., whereas in the group receiving drops at room temperature, the greatest need was observed between 6 p.m. and 12 a.m. On Day 2, both groups showed the highest analgesic consumption between 12 p.m. and 6 p.m. Overall, patients in both groups required the least amount of pain medication during the first quarter of the day on both days.

## 4 DISCUSSION

### 4.1 Synopsis of key findings

Since common drugs used in postoperative pain management after PTK may implicate side effects, we wanted to investigate an easy, safe and inexpensive method of pain reduction. We hypothesised that chilled eye drops might reduce postoperative pain and ocular discomfort.

In this study, we defined a minimal clinically important difference (MCID) of 1 point on the NRS to interpret the relevance of pain differences between groups. As no established MCID exists for postoperative pain following PTK, this threshold was based on clinical experience. Overall, no comparison of primary or secondary end points reached statistical significance. However, there was a trend towards higher pain sensation on postoperative Day 1 using chilled eye drops compared with eye drops at room temperature. Fifty‐three per cent reported pain in the chilled group and 29% in the warm group which was a statistically significant difference. In contrast, there was a clinically significant difference in the percentage of patients reporting severe pain (NRS ≥7), with 2% in the chilled group and 11% in the warm group on Day 2.

As there are currently no published studies defining validated pain thresholds for PTK, we relied on established values from broader postoperative pain research to contextualise our findings. The benchmark for an acceptable postoperative pain sensation might be a score of 33 or less on the VAS (Myles et al., 2017). In the chilled group, 83% had a VAS below this benchmark, comparable to 84% in the warm group on postoperative Day 1. On Day 2, these values were 74% and 67%, respectively.

Studies have found a peak of the pain level on the first postoperative day after PRK (Steigleman et al., 2023). Symptoms diminish after approximately 60–72 h along with epithelial healing (Baumeister et al., 2009; Sobas et al., 2017). To date, there are no trials evaluating pain after PTK. In our study, there was a first peak 2 h after surgery in both groups with participants in the warm group reporting more pain than in the chilled group. It should be noted that at this time point, there was still no difference between the groups, since the administration of eye drops had not commenced until 2 h after surgery. In the chilled group, pain remained almost stable on postoperative Days 1, 2 and 3. In contrast, pain within the warm group was higher on Day 2 (median NRS 2, VAS 19) compared to Day 1 (median NRS 1, VAS 4). On Day 3, there was a decrease in pain sensation in the warm group, so that pain levels were similar between both groups. In the chilled group, patients required additional analgesics earlier in the postoperative course than in the warm group.

The additional need for painkillers is not always congruent with pain perception. In a study by Sobas et al., only 40% asked for analgesics although 97% indicated having pain after advanced surface ablation (Sobas et al., 2015). This was comparable to our results after PTK. Overall, 88% had pain (NRS ≥2) in the chilled and warm group whereas only 49% and 43% needed rescue medication, respectively.

In general, our results might be valid for other clinical situations namely patients with corneal epithelial defects either spontaneous, traumatic or iatrogenic. The effect of chilled eye drops on pain sensation might be less relevant in conditions where the corneal sensitivity is reduced, like neurotrophic keratopathy, after herpetic keratitis or after prior ocular surgery, especially corneal interventions.

The results of the exploratory subgroup analyses are considered hypothesis‐generating and should be interpreted with caution. Women exhibit greater susceptibility to pain stimuli than men in general (Fillingim, 2000). In contrast, after PRK and advanced surface ablation, no gender difference in pain was revealed (Garcia, de Andrade, et al., 2016; Garcia, Torricelli, et al., 2016; Sobas et al., 2017). A direct comparison between male and female patients was not performed in our study. Instead, pain perception with chilled versus eye drops at room temperature was analysed separately within the male and female subgroups. On Day 1, male patients reported higher pain levels with chilled compared to warm eye drops, whereas the difference between groups was less pronounced among female patients. On Day 2, female patients reported more pain in the warm compared to the chilled eye drops group, while male patients had slightly higher pain levels in the chilled group. No significant differences were observed in the subgroup analysis according to age. It was noted, however, that younger patients reported more pain compared to those aged over 40 years. In our study, all patients had a maximum ablation depth of 10 μm. Differentiation by extent of photoablation was beyond the scope of our primary research question. Due to manual abrasion, every patient had a corneal erosion which is the primary reason for postoperative pain. In the subgroups according to ablation depth, no difference was observed between the chilled and the warm group. Future studies are needed to explore potential differences in pain perception by sex, age and ablation depth.

### 4.2 Consideration of possible mechanisms

Application of cold is an effective approach to manage pain. In the field of ophthalmology, chilled eye drops and a cooling mask were shown to reduce the basal corneal sensation in general (Fujishima et al., 1997).

Cooling during laser application is not supposed to have a relevant effect on postoperative pain sensation. With modern laser systems, thermal damage during photoablation is minimised, with a mean maximum temperature of 35.6°C using the SCHWIND AMARIS 750S excimer laser with flying spot technology (Haber‐Olguin et al., 2021). Low temperature decreases metabolism and reduces the release of pain mediators (Kitazawa et al., 1999). Therefore, cooling the cornea after surgery might lead to a reduced inflammatory response and corneal sensation. The underlying molecular mechanisms are complex. Activation of transient receptor potential melastatin family member 8 (TRPM8) and other TRP channels seems to play an important role. Immediate activation of TRPM8 by cooling after nerve injury is supposed to result in an instant reduction of inflammatory response with prolonged effects on pain relief (Shetty et al., 2023).

Although our study focuses specifically on PTK, we assume that the molecular mechanisms underlying pain perception are comparable to those in PRK. Chilled eye drops reduce the surface temperature instantly but the duration of this effect is uncertain. Based on our results, we assume that the cooling effect of eye drops does not last long enough to lead to a relevant reduction of inflammatory response in the postoperative period, since on postoperative Day 1, there was no pain reduction compared with eye drops at room temperature. The reason for even greater pain sensation using chilled eye drops remains unclear.

In contrast, severe pain was less in the chilled eye drops group on the second postoperative day. This may be due to faster epithelial healing, although epithelial closure time was not recorded in this study and this assumption should be regarded as hypothesis‐generating.

All patients received multiple perioperative cooling modalities, including chilled BSS and a refrigerated bandage contact lens, irrespective of randomisation. This likely reduced the between‐group temperature gradient and may have diluted the specific effect of chilled postoperative drops. However, our primary objective was to evaluate the effect of eye drop temperature on pain perception. Therefore, we focused exclusively on the temperature of the postoperative eye drops, while keeping the remainder of the perioperative regimen consistent across all patients. A broader comparison including chilled versus warm BSS, bandage contact lenses and eye drops may yield additional insights and is a potential direction for future research.

### 4.3 Comparison with findings from other studies

The use of chilled BSS, chilled bandage contact lenses or cold patches has only been evaluated after PRK, but the effectiveness of these methods remains controversial. Results from studies evaluating PRK are not directly comparable to those of our research. First of all, the study populations in these trials are younger than in our PTK study. Secondly, manual abrasion during PTK has a diameter of more than 8 mm and is less standardised compared to the abrasion performed during PRK. Finally, the ablation depth of PRK is higher and less homogeneous between individuals compared to PTK. In our study, the maximum ablation depth was 10 μm.

Most of the studies were conducted with older laser technologies. Modern laser machines use smaller laser spots delivering less energy to the cornea which contributes to a reduced increase in temperature and consequently less release of inflammatory mediators (Zarei‐Ghanavati et al., 2017). In a study by Kitazawa et al. from 1999, the effect of cooled PRK was evaluated in 38 patients with high myopia. They continuously irrigated the cornea with chilled BSS 3 min before and after surgery and placed a ring of polymer soaked with cooled BSS on the cornea during the laser ablation. On the first and second postoperative days, pain on VAS was statistically lower after the use of chilled BSS compared with BSS at room temperature (Kitazawa et al., 1999).

Some patients reported flushing the cornea with chilled BSS at the end of the PRK to be uncomfortable. For this reason, Neuffer et al. hypothesised this step to be unnecessary. They found no difference in pain sensation compared with flushing with BSS at room temperature during the first 5 postoperative days. Therefore, they recommended BSS at room temperature in an effort to reduce intraoperative discomfort without any impact on postoperative pain (Neuffer et al., 2013). Similarly, based on the VAS, verbal rating scale and Short‐Form McGill Pain Questionnaire, chilled BSS in combination with a chilled bandage contact lens had no positive effect on postoperative pain or epithelial healing after PRK in a contralateral eye study (Zarei‐Ghanavati et al., 2017). In another study, the use of chilled bandage contact lenses compared with lenses at room temperature reduced pain perception on the first postoperative day after PRK or crosslinking. No difference in epithelial healing time was found (Shetty et al., 2023).

The aforementioned studies used the effect of cold only immediately after the laser procedure and did not continue cooling during postoperative management. Zeng et al. compared flushing the cornea with cold BSS at the end of the PRK and wearing a cold patch for 24 h postoperative. During the first postoperative day, wearing the cold patch was associated with reduced eyelid oedema, corneal hyperaemia and analgesic use, without affecting corneal healing (Zeng et al., 2015). Obviously, participants could not be blinded. This is why a certain placebo effect in regard to pain sensation should be kept in mind.

To date, differences between chilled eye drops and eye drops at room temperature in regard to pain sensation in the postoperative period after PRK or PTK have not been evaluated.

## 5 LIMITATIONS

One limitation of this study is the potential for performance and detection bias, as participants were likely able to perceive the temperature of the administered eye drops, despite being blinded to the storage conditions. Due to the nature of the intervention, full blinding was not feasible, which may have influenced subjective pain reporting. Furthermore, eye drops were administered not by study nurses, but by the routine nursing staff on the ward.

Pain sensation is a complex biopsychosocial process influenced by several individual factors, for example, age, ethnicity and individual approach to pain (Fillingim, 2000; Orhan et al., 2018; Sobas et al., 2017). As we did not conduct a contralateral eye study, we could not account for inter‐individual variabilities. However, contralateral eye trials also have the disadvantage of being influenced by processes in the central nervous system. Owing to the large sample size we do not expect any influence on the results since intra‐individual disparities are evenly distributed in both groups. Differences in pain perception and attitude have been demonstrated in different populations (Orhan et al., 2018). Thus, the generalisability of our study is restricted because especially Caucasian participants were part of our trial. In general, our findings should be interpreted with caution, as all procedures were performed by a single surgeon at a single centre, nearly all participants were Caucasian and ablation depths were shallow (≤10 μm). These factors may limit generalisability to other PTK indications, patient populations and surgical settings.

## 6 CONCLUSIONS

This is the first study analysing differences in postoperative pain sensation in the early postoperative period after PTK using either chilled eye drops or eye drops at room temperature. The use of chilled eye drops is not beneficial compared with eye drops at room temperature on the first postoperative day. Even more pain was reported using chilled eye drops. On the second postoperative day, pain seems to be reduced with chilled compared with warm eye drops to a clinically significant degree, but without reaching statistical significance. Faster epithelial healing may contribute to this effect, but as it was not measured, this remains hypothesis‐generating and warrants future investigation.

## CONFLICT OF INTEREST STATEMENT

K. P. K. Lecturing for Oculus. T. K. Consultant, Research and Lecturing for Alcon, Oculus, Schwind, Staar. Consultant and Lecturing for Tarsus, Ziemer. Research and Lecturing for Teleon Surgical. Consultant for Abbvie, Geuder, LensGen, Santen, Stadapharm, Thieme, Zeiss Meditec. Lecturing for Allergan, Bausch and Lomb, Johnson and Johnson, MedUpdate, streamedup. C. M. K.‐W., J. B., M. L. B., P. D., K. V.‐T., I. S. have no conflict of interest to declare.

## Data Availability

The anonymised data that support the findings of this study are available from the corresponding author upon reasonable request.

## References

[aos70033-bib-0001] Baumeister, M. , Bühren, J. , Ohrloff, C. & Kohnen, T. (2009) Corneal re‐epithelialization following phototherapeutic keratectomy for recurrent corneal erosion as in vivo model of epithelial wound healing. Ophthalmologica, 223, 414–418. 19648776 10.1159/000230880

[aos70033-bib-0002] Belmonte, C. , Acosta, M.C. & Gallar, J. (2004) Neural basis of sensation in intact and injured corneas. Experimental Eye Research, 78, 513–525. 15106930 10.1016/j.exer.2003.09.023

[aos70033-bib-0003] Fillingim, R.B. (2000) Sex, gender, and pain: women and men really are different. Current Review of Pain, 4, 24–30. 10998712 10.1007/s11916-000-0006-6

[aos70033-bib-0004] Förster, W. , Ratkay, I. , Krueger, R. & Busse, H. (1997) Topical diclofenac sodium after excimer laser phototherapeutic keratectomy. Journal of Refractive Surgery, 13, 311–313. 9183765 10.3928/1081-597X-19970501-20

[aos70033-bib-0005] Fujishima, H. , Yagi, Y. , Shimazaki, J. & Tsubota, K. (1997) Effects of artificial tear temperature on corneal sensation and subjective comfort. Cornea, 16, 630–634. 9395871

[aos70033-bib-0006] Gallar, J. , Acosta, M.C. , Gutiérrez, A.R. & Belmonte, C. (2007) Impulse activity in corneal sensory nerve fibers after photorefractive keratectomy. Investigative Ophthalmology & Visual Science, 48, 4033–4037. 17724184 10.1167/iovs.07-0012

[aos70033-bib-0007] Garcia, R. , de Andrade, D.C. , Teixeira, M.J. , Nozaki, S.S. & Bechara, S.J. (2016) Mechanisms of corneal pain and implications for postoperative pain after laser correction of refractive errors. The Clinical Journal of Pain, 32, 450–458. 27504514 10.1097/ajp.0000000000000271

[aos70033-bib-0008] Garcia, R. , Torricelli, A.A.M. , Mukai, A. , Pereira, V.B.P. & Bechara, S.J. (2016) Predictors of early postoperative pain after photorefractive keratectomy. Cornea, 35, 1062–1068. 27124781 10.1097/ICO.0000000000000859

[aos70033-bib-0009] Gewandter, J.S. , Eisenach, J.C. , Gross, R.A. , Jensen, M.P. , Keefe, F.J. , Lee, D.A. et al. (2019) Checklist for the preparation and review of pain clinical trial publications: a pain‐specific supplement to CONSORT. Pain Rep, 4, e621. 28989992 10.1097/PR9.0000000000000621 PMC5625298

[aos70033-bib-0010] Haber‐Olguin, A. , Polania‐Baron, E.J. , Trujillo‐Trujillo, F. & Graue Hernandez, E.O. (2021) Thermographic behavior of the cornea during treatment with two excimer laser platforms. Translational Vision Science & Technology, 10, 27. 10.1167/tvst.10.9.27 PMC8399240 34427627

[aos70033-bib-0011] Kitazawa, Y. , Maekawa, E. , Sasaki, S. , Tokoro, T. , Mochizuki, M. & Ito, S. (1999) Cooling effect on excimer laser photorefractive keratectomy. Journal of Cataract and Refractive Surgery, 25, 1349–1355. 10511934 10.1016/s0886-3350(99)00207-2

[aos70033-bib-0012] Marfurt, C.F. , Cox, J. , Deek, S. & Dvorscak, L. (2010) Anatomy of the human corneal innervation. Experimental Eye Research, 90, 478–492. 20036654 10.1016/j.exer.2009.12.010

[aos70033-bib-0013] Moher, D. , Hopewell, S. , Schulz, K.F. , Montori, V. , Gotzsche, P.C. , Devereaux, P.J. et al. (2010) CONSORT 2010 explanation and elaboration: updated guidelines for reporting parallel group randomised trials. BMJ, 340, c869. 20332511 10.1136/bmj.c869 PMC2844943

[aos70033-bib-0014] Müller, L.J. , Marfurt, C.F. , Kruse, F. & Tervo, T.M.T. (2003) Corneal nerves: structure, contents and function. Experimental Eye Research, 76, 521–542. 12697417 10.1016/s0014-4835(03)00050-2

[aos70033-bib-0015] Myles, P.S. , Myles, D.B. , Galagher, W. , Boyd, D. , Chew, C. , MacDonald, N. et al. (2017) Measuring acute postoperative pain using the visual analog scale: the minimal clinically important difference and patient acceptable symptom state. British Journal of Anaesthesia, 118, 424–429. 28186223 10.1093/bja/aew466

[aos70033-bib-0016] Neuffer, M.C. , Khalifa, Y.M. , Moshirfar, M. & Miffin, M.D. (2013) Prospective comparison of chilled versus room temperature saline irrigation in alcohol‐assisted photorefractive keratectomy. Nepalese Journal of Ophthalmology, 5, 154–160. 24172548 10.3126/nepjoph.v5i2.8706

[aos70033-bib-0017] Orhan, C. , Van Looveren, E. , Cagnie, B. , Mukhtar, N.B. , Lenoir, D. & Meeus, M. (2018) Are pain beliefs, cognitions, and behaviors influenced by race, ethnicity, and culture in patients with chronic musculoskeletal pain: a systematic review. Pain Physician, 21, 541–558. 30508984

[aos70033-bib-0018] Shetty, R. , Shetty, N. , Shirodkar, S. , Ashok, N. , Sethu, S. , Ghosh, A. et al. (2023) Cold bandage contact lens use reduces post‐photorefractive keratectomy or corneal collagen‐crosslinking pain perception in patients. Indian Journal of Ophthalmology, 71, 1855–1861. 37203044 10.4103/IJO.IJO_2757_22 PMC10391479

[aos70033-bib-0019] Sobas, E.M. , Videla, S. , Maldonado, M.J. & Pastor, J.C. (2015) Ocular pain and discomfort after advanced surface ablation: an ignored complaint. Clinical Ophthalmology, 9, 1625–1632. 26379419 10.2147/OPTH.S86812 PMC4567230

[aos70033-bib-0020] Sobas, E.M. , Videla, S. , Vázquez, A. , Fernández, I. , Maldonado, M.J. & Pastor, J.‐C. (2017) Pain perception description after advanced surface ablation. Clinical Ophthalmology, 11, 647–655. 28435216 10.2147/OPTH.S134542 PMC5391165

[aos70033-bib-0021] Steigleman, W.A. , Rose‐Nussbaumer, J. , Al‐Mohtaseb, Z. , Santhiago, M.R. , Lin, C.C. , Pantanelli, S.M. et al. (2023) Management of Pain after photorefractive keratectomy: a report by the American Academy of ophthalmology. Ophthalmology, 130, 87–98. 36207168 10.1016/j.ophtha.2022.07.028

[aos70033-bib-0022] Zarei‐Ghanavati, S. , Nosrat, N. , Morovatdar, N. , Abrishami, M. & Eghbali, P. (2017) Efficacy of corneal cooling on postoperative pain management after photorefractive keratectomy: a contralateral eye randomized clinical trial. Journal of Current Ophthalmology, 29, 264–269. 29270472 10.1016/j.joco.2017.04.004 PMC5735231

[aos70033-bib-0023] Zeng, Y. , Li, Y. & Gao, J.‐H. (2015) Application of cold patch in relieving pain after transepithelial photorefractive keratectomy. Pain Research & Management, 20, 195–198. 25992866 10.1155/2015/850245 PMC4532205

